# ﻿An annotated checklist and the first Red List of harvestmen (Opiliones) of Slovakia

**DOI:** 10.3897/zookeys.1230.134516

**Published:** 2025-03-05

**Authors:** Juraj Litavský, Slavomír Stašiov, Ivan Mihál, Martin Šalkovič

**Affiliations:** 1 Department of Environmental Ecology and Landscape Management, Faculty of Natural Sciences, Comenius University, Ilkovičova 6, SK 842 15 Bratislava, Slovakia Comenius University Bratislava Slovakia; 2 Department of Biology and General Ecology, Faculty of Ecology and Environmental Sciences, Technical University in Zvolen, T. G. Masaryka 24, SK 960 01 Zvolen, Slovakia Technical University in Zvolen Zvolen Slovakia; 3 Institute of Forest Ecology, Slovak Academy of Sciences, Štúrova 2, SK 960 53 Zvolen, Slovakia Slovak Academy of Sciences Zvolen Slovakia

**Keywords:** Arachnida, diversity, IUCN categories, species conservation, zoogeography

## Abstract

Although harvestmen are a significant group of terrestrial invertebrates, their effective protection has not been ensured in Slovakia to this day. Not a single species belonging to the order Opiliones has been included in the “Red Data Book of threatened and rare plant and animal species of the Czech and Slovak Federal Republic”. Harvestmen have also not been included in the Red List of plants and animals of Slovakia. Since a new Red Data Book of invertebrates of Slovakia is currently under production, a checklist and the first Red List of harvestmen of Slovakia were prepared. Thirty-five species of harvestmen were identified based on the analysis of all records published since 1873 and our unpublished records. A total of 5,254 records of harvestman species from 2,772 locations were analysed across 318 grid cells, each measuring 10 × 10 km. Two species were recorded exclusively in the Pannonian and six in the Alpine biogeographical region, while the remaining species occurred in both regions. All 35 species were assessed based on the current IUCN Red List criteria, categorising them as follows: Critically Endangered (*Holoscotolemonjaqueti*), Endangered (*Sirocarpaticus*), Vulnerable (*Gyastitanus*, *Ischyropsalismanicata*, *Paranemastomakochii*, *Paranemastomaquadripunctatum*), Near Threatened (*Laciniusdentiger*, *Laciniushorridus*, *Opilioparietinus*, *Platybunuspallidus*), Least Concern (22 species), Data Deficient (2), and Not Evaluated (1). A brief overview of the history of Opiliones research in Slovakia is also provided.

## ﻿Introduction

The order Opiliones consists of 6,757 species belonging to 1,704 genera, 71 families, and 4 suborders: Cyphophthalmi, Dyspnoi, Eupnoi, and Laniatores (Kury et al. 2024), to which an extinct suborder Tetrophthalmi was also assigned (Garwood et al. 2014). They are distributed on all continents except Antarctica. Their estimated diversity comprises more than 10,000 species (Pinto-Da-Rocha et al. 2005; Boyer et al. 2007).

Regarding the history of opiliofauna research in Slovakia, Sørensen’s work (Sørensen 1873) represents the first and oldest publication providing data on harvestmen at the territory of today’s Slovakia. Following that, Herman’s work (Herman 1879) focused on findings of harvestmen around Bratislava. Petricskó (1892) in his monograph, documented the presence of seven species of harvestmen from the vicinity of Banská Štiavnica. Another study containing information on harvestmen from areas of Bratislava, Bardejov, Nitrianske Pravno, Starý Smokovec, and Popradské pleso was conducted by Daday (1918). Among the earliest works dedicated to the issue of the Slovak opiliofauna are also studies by Roewer (1923), Dudich (1928), Kolosváry (1929, 1933), Bartoš (1939a, 1939b), Kratochvíl (1933, 1934, 1939), and Dudich et al. (1940). A significant contribution to the knowledge of the opiliofauna of Slovakia was made by V. Šilhavý (Šilhavý 1949, 1950, 1956, 1966, 1968a, 1968b, 1970, 1972, 1974, 1981), who published findings on the distribution, ecology, and ontogeny of our species. He devoted himself to the taxonomy of harvestmen and resolved several ambiguities in their systematics, thereby advancing the research on Opiliones at the pan-European level. His most significant work (Šilhavý 1956) contains descriptions of 64 species of harvestmen recorded in the territory of the former Czechoslovakia, or the author assumed their distribution there. The first Slovak authors who studied the fauna of harvestmen in our country were Ferianc (1949), Lác (1957, 1990), Ložek and Gulička (1955), Gulička (1957, 1985). Jasenák (1972) also conducted studies on harvestmen in the Devínska Kobyla Nature Reserve. Hroznár (1981) described the fauna of harvestmen in the Kriváň – part of the Malá Fatra mountain. Košel (1984, 1994) published records of the species *Ischyropsalismanicata* in some caves in Slovakia. Majzlan and Hazuchová (1997) conducted research on opiliofauna in various locations in the floodplain forests of Podunajská nížina (Danubian Lowland). In the 1990s, Mašán and Mihál (1993) and Mašán (1998) dedicated himself to the research of harvestmen in Slovakia. Since the 1990s, B. Astaloš, I. Mihál, and S. Stašiov have been the most active contributors to the study of the harvestman fauna. Their collaboration, in partnerships with other specialists in this group of organisms has led to significant progress in understanding the harvestman fauna of Slovakia, particularly in terms of faunistics, the ecology of individual species, and their bioindication potential. P. Maršalek (Maršalek 2001), A. Šestáková (Šestáková and Mihál 2014), and J. Litavský (Litavský et al. 2018, 2019) later joined those authors.

The assembly of the Red List of harvestmen of Slovakia was prompted by the requirement to provide a legislative basis for a more efficient and effective protection of threatened and rare harvestman species, which is based on current knowledge about their distribution, ecology, and the threat of their populations in Slovakia.

## ﻿Material and methods

### ﻿Study area

**Figure 1.**
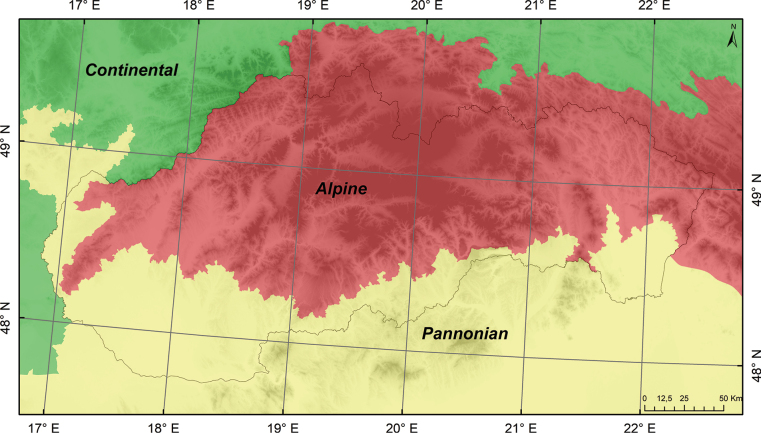
Biogeographical regions of Slovakia.

The research was conducted in Slovakia, a Central European country with an area of 49,035 km^2^. The lowest point of the country, at an elevation of 94 meters a.s.l., is located on the Bodrog River on the Slovak-Hungarian border. On the contrary, the highest point in Slovakia is Gerlach Peak in the High Tatras, reaching an altitude of 2,655 meters a.s.l. Slovakia is located in the central part of the Carpathian Arc, which is divided into the Western, Eastern, and Southern Carpathians, with its territory primarily covered by the Western Carpathians, featuring diverse mountain ranges, basins, and lowlands. A significant geological region of the Western Carpathians is the Slovenské rudohorie (Slovak Ore Mountains), which was formed through complex tectonic processes associated with folding, volcanism, and erosion during older geological periods. From the perspective of the biogeographical division of Europe, Slovakia is located in two regions: Alpine and Pannonian (Fig. 1).

The climate in Slovakia is quite diverse, due to its geographical location and varied terrain. Most of the country experiences a temperate continental climate, characterised by cold winters and warm summers, with milder winters and warmer summers in the lower elevations. The High Tatras and other higher mountain ranges have a cooler climate, with long winters and shorter, cooler summers. The Pannonian Plain has a warmer climate, with drier summers and mild winters. Atmospheric precipitation in the territory of Slovakia is more influenced by the geographical location of the territory, altitude, wind direction or leeward direction of the territory to the prevailing flow, which brings moist air masses. The average yearly temperature is ~ 10 °C, with January being the coldest month, averaging -3 °C. In contrast, July and August see the highest averages, reaching 26 °C. Annual precipitation amounts to ~ 650 mm, with July being the wettest month, receiving around 73 mm of rainfall over 11 days. The northern mountainous regions of Slovakia experience cooler and wetter conditions, whereas the southern areas are characterised by a warmer and drier climate (Faško and Šťastný 2002; Lapin et al. 2002; Bochníček and Hrušková 2015).

The landscape of Slovakia is characterised by great diversity, including mountain ranges, basins, lowlands, rivers, and lakes. The lowlands are located mainly in the south and southeast of Slovakia. The most fertile and well-known is the Danubian Lowland, while other lowlands include the East Slovak Lowland and the Záhorie Lowland. The mountain ranges include the Low Tatras and the Greater and Lesser Fatras, and the mountains feature the High Tatras. Slovakia has a wide variety of soil types, resulting from various geological and climatic conditions that also affect the diversity of the flora. In lower elevations, where chernozem and brown soils dominate, there are mainly meadows and agricultural landscape. In the middle and higher elevations, where acid podzolic soils and forest soils prevail, forests predominate, dominated by beech, oak, spruce, and fir. In mountainous areas, where peat and wet soils are found, alpine and subalpine vegetation is typical, including various types of vegetation adapted to a colder and wetter environment. This relationship between soil and flora creates various ecosystems that are characteristic of individual regional conditions in Slovakia (Lukniš 1972).

### ﻿Data sampling and harvestmen mapping

We analysed all available data on harvestmen in Slovakia, including published works up to January 2024 and unpublished records from the first three authors. Building on the comprehensive work “Harvestmen of Slovakia” (Stašiov 2004), which covered data until the end of 2003, we incorporated this earlier information and added distribution records from the next 20 years (2004–2024). This allowed us to assess diversity, monitor changes in the opiliofauna, and propose a red list of harvestmen of Slovakia.

To assemble a Red List of Slovak harvestmen and the interpretation of the results using maps, we created map bases with a grid cell size of 10 × 10 km according to Murray (2017). We examined 284 literary data (from 1873 to January 2024), as well as our own previously unpublished data on the occurrence of harvestmen from a total of 2,772 locations within Slovakia. We considered the number of locations where research on harvestmen was conducted – records of harvestmen within each individual grid cell. We also assessed the species richness of harvestmen per individual grid cells covering the territory of Slovakia. Data on harvestmen records from Slovakia were processed and graphically represented for the period from 1873 to the end of 2003, and from the beginning of 2004 to January 2024. The total number of records of harvestmen in Slovakia is 5,254, which shows the total number of species-specific records during 150 years of research on this group of organisms in our country (Table 1). Most data on the occurrence of harvestmen in Slovakia were obtained using pitfall traps, hand collection, and soil and litter sieving. Due to variations in capture methods and study durations (e.g., half-year, annual, or multi-year pitfall studies versus one-day or multi-day hand collection or litter sieving), analysing and comparing the abundance of recorded individuals across these studies was not considered meaningful.

**Table 1.** Checklist of harvestman species recorded in Slovakia up to 2024 with IUCN Red list categories and criteria. Acronyms of Red List categories: CE – Critically Endangered, EN – Endangered, VU – Vulnerable, NT – Near Threatened, LC – Least Concern, DD – Data Deficient, NE – Not Evaluated (for non-native species). Total number of records shows the total number of species-specific records since 1873 to January 2024. Share of records from 2004–2024 [%] – share of records obtained in the years 2004–2024 relative to the total number of records. Share of records until 2004 [%] – share of records obtained until 2004 relative to the total number of records. Number of grid cells occupied – the number of grid cells in which the species has been recorded. BR – biogeographical region; Red List of Austria (Komposch 2009), Red List of the Czech Republic (Bezděčka and Bezděčková 2017) and Red List of Poland (Głowaciński 2002).

	Red List Category	Red List Criteria	Number of records before 2004	Number of records since 2004	Total number of records	Share of records from 2004–2024 [%]	Share of records until 2004 [%]	Number of grid cells occupied	Distribution in the Alpine BR	Distribution in the Pannonian BR	Red List of Austria (2009)	Red List of the Czech Republic (2017)	Red List of Poland (2002)
**Suborder**, family, species													
**Cyphophthalmi Simon, 1879**													
Sironidae Simon, 1879													
*Sirocarpaticus* Rafalski, 1956	EN	B1ab (iii.v)	8	2	10	20.0	80.0	9	10	0			EN
**Eupnoi Hansen & Sørensen, 1904**													
Sclerosomatidae Simon, 1879													
*Astrobunuslaevipes* (Canestrini, 1872)	LC		65	125	190	65.8	34.2	70	102	88	VU		EN
*Gyastitanus* Simon, 1879	VU	B1ab (iii.v)	69	18	87	20.7	79.3	48	87	0	EN		
*Leiobunumlimbatum* Koch, 1861	LC		0	6	6	100.0	0.0	6	6	0	LC		EN
*Leiobunumrotundum* (Latreille, 1798)	LC		44	41	85	48.2	51.8	50	62	23	NT		
*Leiobunumgracile* Thorell, 1876	LC		123	94	217	43.3	56.7	112	194	23	LC		
*Nelimasempronii* Szalay, 1951	LC		8	120	128	93.8	6.3	44	49	79	LC		
Phalangiidae Latreille, 1801													
*Dicranopalpus* sp. Doleschall, 1852	DD		1	0	1	0.0	100.0	1	1	0	LC		
*Egaenusconvexus* (Koch, 1835)	LC		76	142	218	65.1	34.9	79	121	97	VU	NT	EN
*Laciniusdentiger* (Koch, 1848)	NT		38	27	65	41.5	58.5	31	27	38	LC		VU
*Laciniusephippiatus* (Koch, 1835)	LC		154	175	329	53.2	46.8	131	280	49	NT		
*Laciniushorridus* (Panzer, 1794)	NT		64	40	104	38.5	61.5	55	81	23	VU		
*Lophopiliopalpinalis* (Herbst, 1799)	LC		131	96	227	42.3	57.7	103	192	35	LC		
*Mitopusmorio* (Fabricius, 1799)	LC		266	103	369	27.9	72.1	139	339	28	LC		
*Oligolophustridens* (Koch, 1836)	LC		169	148	317	46.7	53.3	131	248	69	LC		
*Opiliocanestrinii* (Thorell, 1876)	NE		2	44	46	95.7	4.3	21	20	26	NE		
*Opiliodinaricus* Šilhavý, 1938	DD		1	0	1	0.0	100.0	1	1	0	NT		
*Opilioparietinus* (De Geer, 1778)	NT		68	14	82	17.1	82.9	59	53	29	EN		
*Opiliosaxatilis* Koch, 1839	LC		56	66	122	54.1	45.9	50	54	68	LC		
*Phalangiumopilio* Linnaeus, 1761	LC		177	138	315	43.8	56.2	122	239	76	LC		
*Platybunusbucephalus* (Koch, 1835)	LC		224	92	316	29.1	70.9	120	301	15	LC		
*Platybunuspallidus* Šilhavý, 1938	NT		37	28	65	43.1	56.9	44	65	0			EN
*Rilaenatriangularis* (Herbst, 1799)	LC		86	155	241	64.3	35.7	109	165	76	LC		
*Zachaeuscrista* (Brullé, 1832)	LC		88	132	220	60.0	40.0	78	150	70		NT	
**Dyspnoi Hansen & Sørensen, 1904**													
Dicranolasmatidae Simon, 1879													
*Dicranolasmascabrum* (Herbst, 1799)	LC		77	83	160	51.9	48.1	76	127	33	EN	VU	
Ischyropsalididae Simon, 1879													
*Ischyropsalismanicata* Koch, 1869	VU	B1ab (i.iii.v)	66	24	90	26.7	73.3	50	85	5		VU	VU
Nemastomatidae Simon, 1871													
*Carinostomaelegans* (Sørensen, 1894)	LC		0	14	14	100.0	0.0	8	0	14			
*Mitostomachrysomelas* (Hermann, 1804)	LC		143	91	234	38.9	61.1	107	177	57	LC		
*Nemastomabidentatumsparsum* Gruber & Martens, 1968	LC		9	42	51	82.4	17.6	10	0	51	NT	VU	
*Nemastomalugubre* (Müller, 1776)	LC		218	167	385	43.4	56.6	161	323	62	EN		
*Paranemastomakochii* (Nowicki, 1870)	VU	B1ab (iii.v)	74	35	109	32.1	67.9	58	105	4		VU	
*Paranemastomaquadripunctatum* (Perty, 1833)	VU	B1ab (i.iii.v)	1	1	2	50.0	50.0	2	2	0	NT		
Trogulidae Sundevall, 1833													
*Trogulusnepaeformis* (Scopoli, 1763)	LC		112	122	234	52.1	47.9	107	191	43	DD		VU
*Trogulustricarinatus* (Linnaeus, 1767)	LC		51	155	206	75.2	24.8	73	112	94	DD		
**Laniatores Thorell, 1876**													
Cladonychiidae Hadži, 1935													
*Holoscotolemonjaqueti* (Corti, 1905)	CR	B1ab (iii.v)	4	4	8	50.0	50.0	2	0	8			

A geodatabase was created in ArcGIS Pro using descriptive data provided by the authors, including location, altitude, slope orientation, and cadastral area. Records lacking precise localisation (e.g., “the Tatras”) or involving questionable species identifications, such as *Nemastomabidentatum* near Bardejov (Sørensen 1873) and *Opiliodinaricus* in Bratislava (Hajková 1995), were excluded from the analysis.

### ﻿Species list and nomenclature

The presented list includes all species of Opiliones known from Slovakia, divided into higher taxonomic units; suborders are indicated in bold, families are underlined, and species are in italics (Table 1). The species richness of harvestmen within individual grid cells is also provided. Since Slovakian harvestman species are not listed on the IUCN Red List, threat categories were determined based on the official criteria of the IUCN Standards and Petitions Committee (IUCN 2022). During our study of harvestmen, we identified specimens using the keys provided by Šilhavý (1956), Martens (1978), and Wijnhoven (2009). The nomenclature of harvestmen follows Kury et al. (2024).

## ﻿Results and discussion

**Figure 2.**
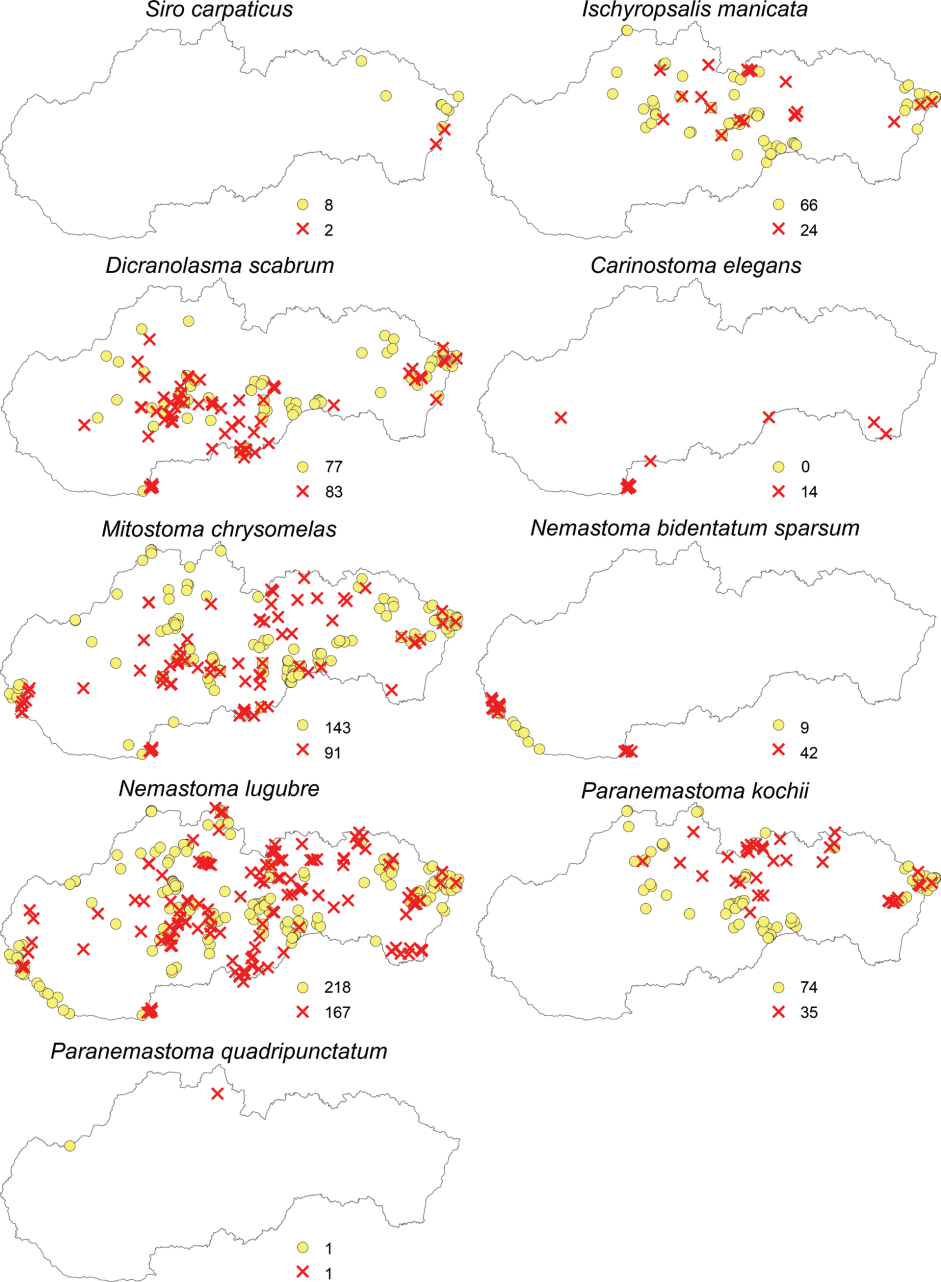
Distribution of harvestmen (Opiliones) in Slovakia belonging to the families Sironidae, Ischyropsalididae, Dicranolasmatidae, and Nemastomatidae (yellow circles represent the locations of individual species recorded before 2004, and red X marks indicate the locations of species recorded since 2004; the numbers next to these symbols represent the total number of records).

**Figure 3.**
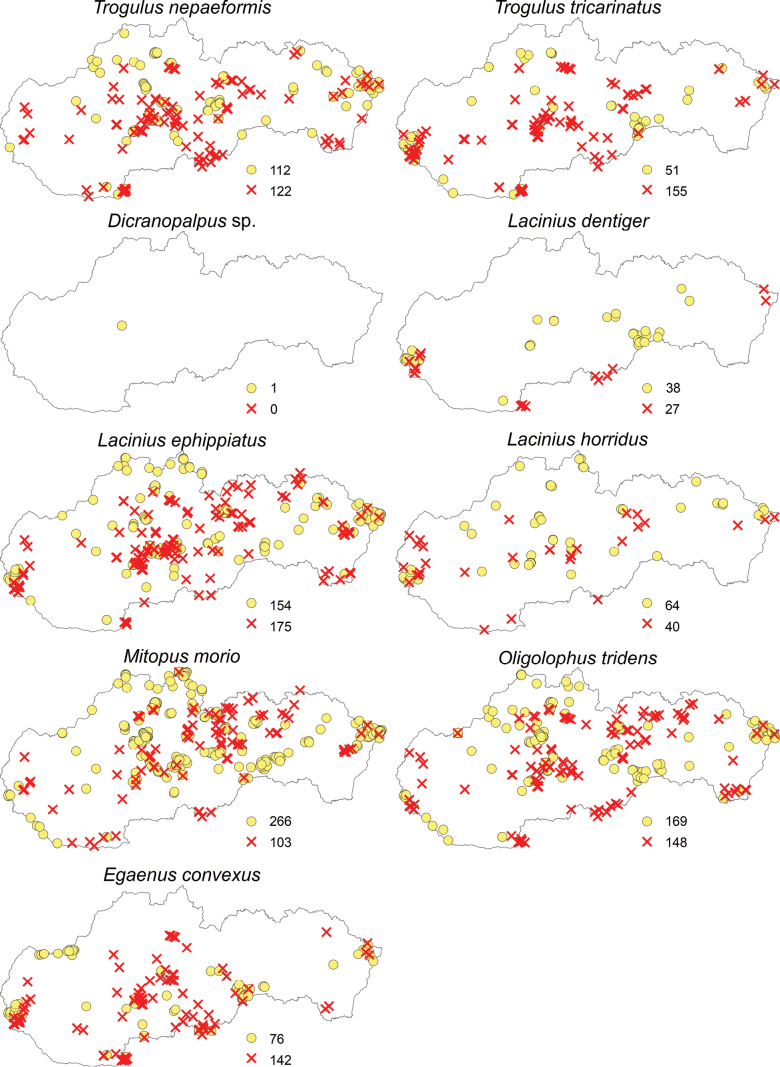
Distribution of harvestmen (Opiliones) in Slovakia belonging to the families Trogulidae and Phalangiidae (yellow circles represent the locations of individual species recorded before 2004, and red X marks indicate the locations of species recorded since 2004; the numbers next to these symbols represent the total number of records).

**Figure 4.**
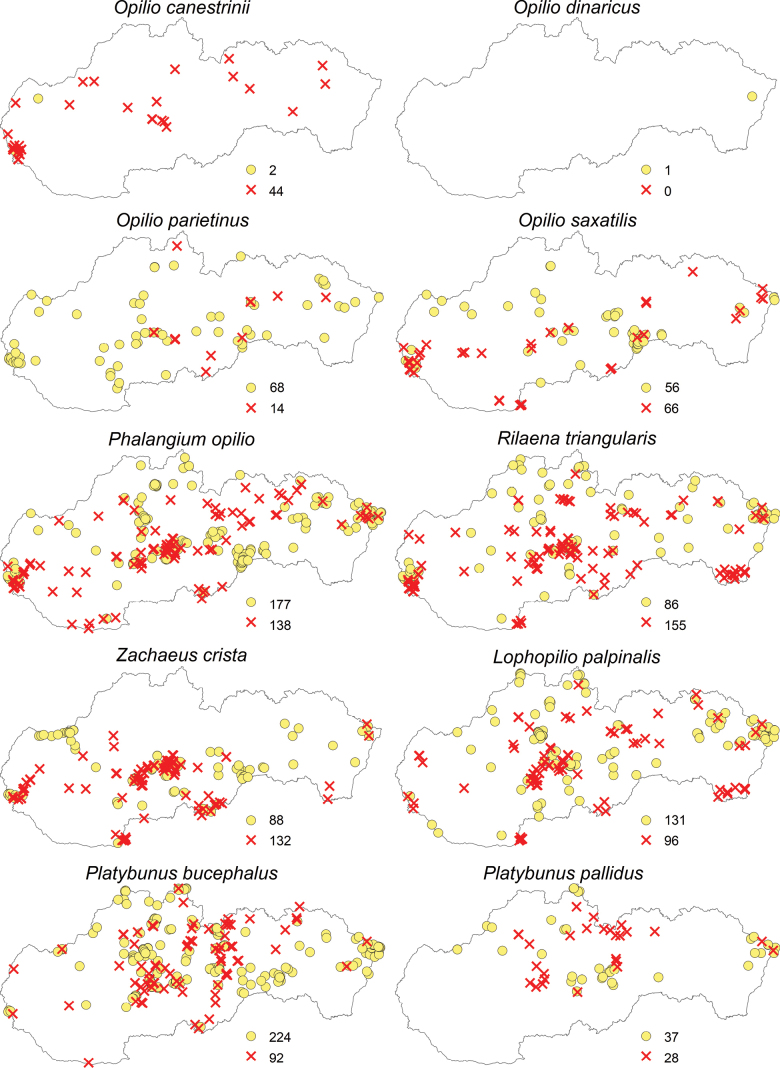
Distribution of harvestmen (Opiliones) in Slovakia belonging to the family Phalangiidae (yellow circles represent the locations of individual species recorded before 2004, and red X marks indicate the locations of species recorded since 2004; the numbers next to these symbols represent the total number of records).

**Figure 5.**
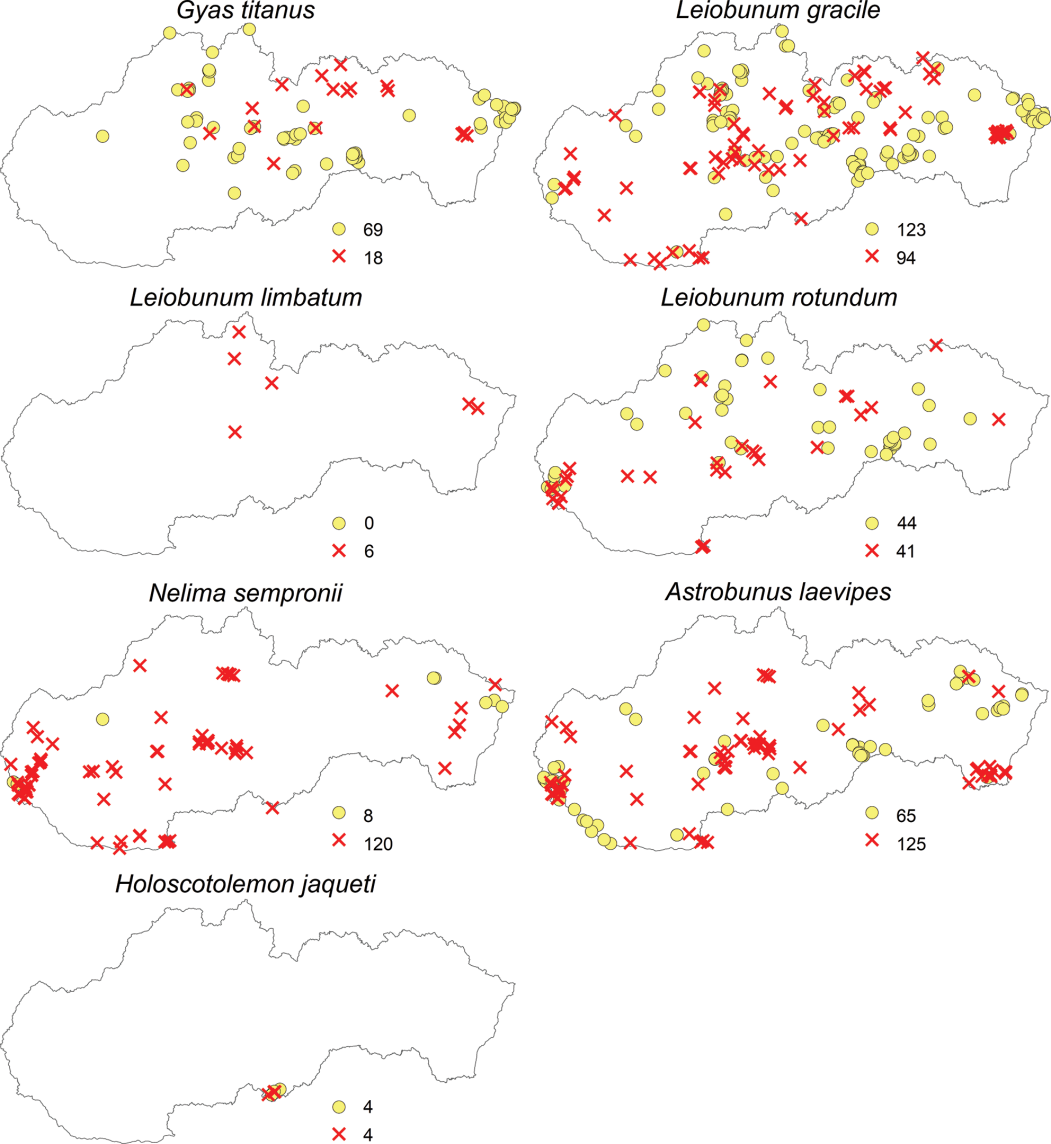
Distribution of harvestmen (Opiliones) in Slovakia belonging to the families Sclerosomatidae and Cladonychiidae (yellow circles represent the locations of individual species recorded before 2004, and red X marks indicate the locations of species recorded since 2004; the numbers next to these symbols represent the total number of records).

Regarding the opiliofauna of Slovakia, 35 species from eight families and four suborders have been recorded (Table 1). The suborders Cyphophthalmi (family Sironidae) and Laniatores (family Cladonychiidae) are represented in Slovakia by a single species each (Figs 2–5). From the suborder Dyspnoi, 10 species from 4 families are known in Slovakia. Their distribution in Slovakia is shown in Figs 2, 3. There is one species each documented in our territory from the families Dicranolasmatidae and Ischyropsalididae, while there are two species from the family Trogulidae and four genera with six species from the family Nematostomatidae. More than half of the harvestman fauna in Slovakia is in the suborder Eupnoi: the family Phalangiidae is represented by 11 genera with 17 species (Figs 3, 4), and the family Sclerosomatidae includes four genera with six species (Fig. 5). The genus *Dicranopalpus* is known from Slovakia (Vtáčnik Mountains) only from immature individuals, which do not allow for reliable species identification.

**Figure 6.**
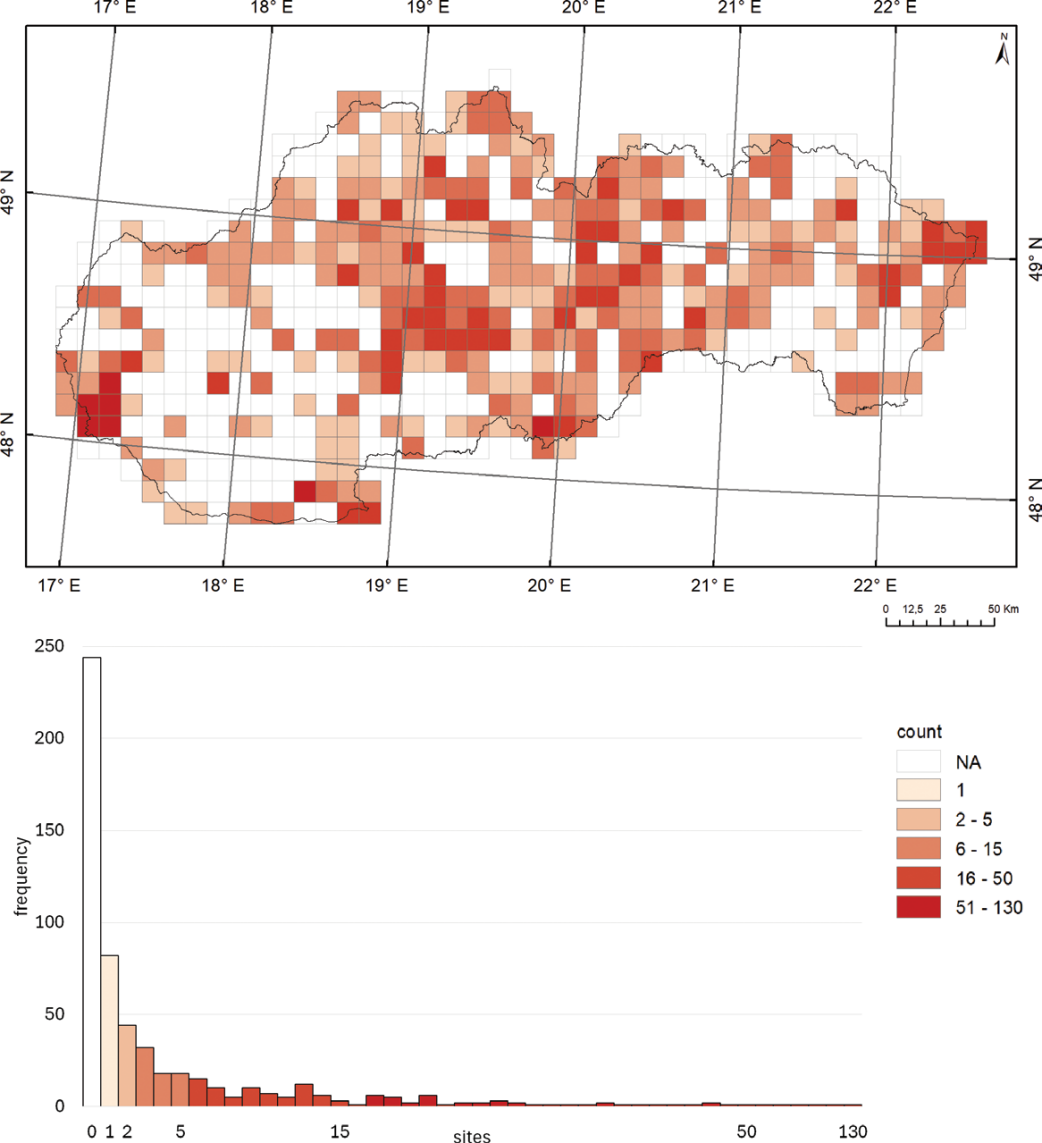
The frequency of sampled sites within 10 × 10 km grid cells in Slovakia, categorised into six levels (*n* = 562 grid cells; NA – not applicable).

So far, 56.6% of the 562 grid cells (10 × 10 km) covering the Slovak territory have been examined at least at one site (Fig. 6). No harvestmen surveys have been conducted in 244 grid cells so far. Of a total of 2,772 studied localities, there is one record per cell in 82 grid cells, 2–5 records per cell in 112 grid cells, 6–15 records per cell in 73 grid cells, 16–50 records per cell in 44 grid cells, and 51–130 records per cell were documented in 7 grid cells covering the territory of Slovakia.

**Figure 7.**
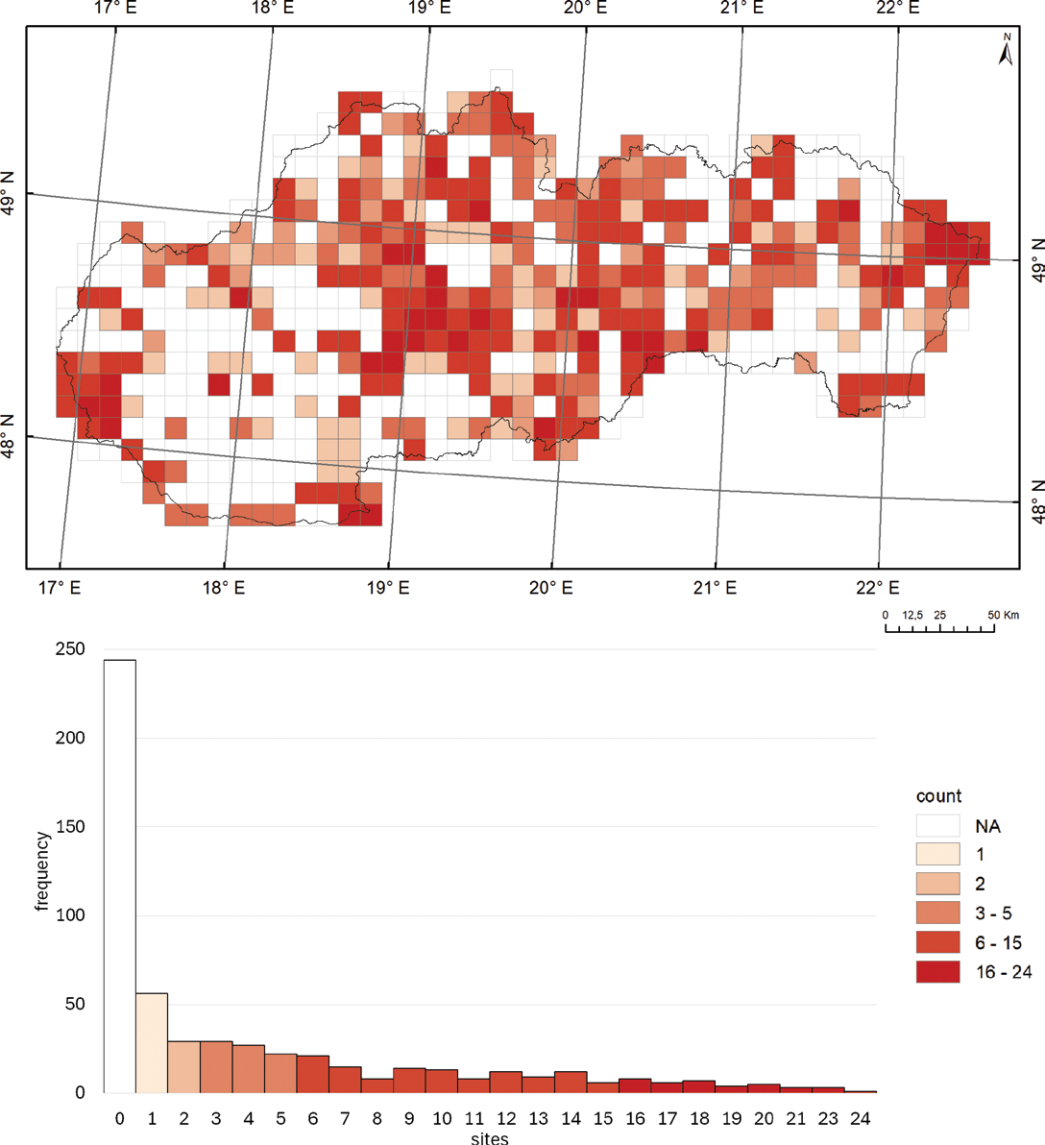
Number of recorded species within 10 × 10 km grid cells in Slovakia. The histogram illustrates the frequency of counts categorised into six levels (*n* = 562 grid cells; NA – not applicable).

Regarding the species richness of harvestmen within the grid cells of Slovakia, one species per cell was recorded in 56 grid cells, two species per cell in 29 grid cells, 3–5 species per cell in 78 grid cells, 6–15 species per cell in 118 grid cells, and 16–24 species per cell in 37 grid cells across the territory of Slovakia (Fig. 7).

Based on the IUCN Red List criteria (IUCN 2022), we assessed all 35 species. The species were red-listed as follows: one Critically Endangered – CE (*Holoscotolemonjaqueti*), one Endangered – EN (*Sirocarpaticus*) and four Vulnerable – VU (*Gyastitanus*, *Ischyropsalismanicata*, *Paranemastomakochii*, *Paranemastomaquadripunctatum*). Four species were assessed as Near Threatened – NT, (*Laciniusdentiger*, *Laciniushorridus*, *Opilioparietinus*, *Platybunuspallidus*), 22 as Least Concern – LC, two as Data Deficient – DD, and one Not Evaluated – NE for a non-native species – *Opiliocanestrinii* (Table 1).

### ﻿Comments on the distribution and ecology of the most endangered species of harvestmen in Slovakia

**Figure 8.**
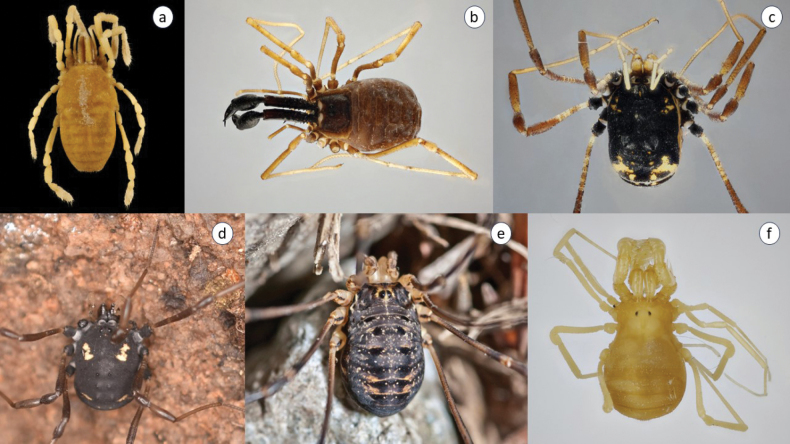
Critically endangered, endangered, and vulnerable species of harvestmen in Slovakia **a***Sirocarpaticus* (photo by A. Christophoryová) **b***Ischyropsalismanicata* (photo by A. Christophoryová) **c***Paranemastomakochii* (photo by A. Christophoryová) **d***Paranemastomaquadripunctatum* (photo by: O. Machač) **e***Gyastitanus* (photo by A. Šestáková) **f***Holoscotolemonjaqueti* (photo by D. Selnekovič).

We have classified six species of harvestmen (Opiliones) in this group (Fig. 8).

#### ﻿Cyphophthalmi Simon, 1879

**Sironidae Simon, 1879**

***Sirocarpaticus* Rafalski, 1956**

*Sirocarpaticus* (Rafalski, 1956): 49–52 (Poland, Bieszczady). Mašán 1998: 650. Mašán 2005: 28. Mihál et al. 2003: 129. Mihál and Mašán 2006: 92. Stašiov et al. 2003a: 263, 265.

**Current distribution in Europe.** This species has a relatively small known range and was described in the second half of the 20^th^ century from southeastern Poland (Rafalski 1956). In addition to Poland, it has been found in northeastern Slovakia. It is highly likely that *S.carpaticus* also occurs in Ukraine, but it has not yet been scientifically confirmed there.

**Current distribution in Slovakia.** In Slovakia, *S.carpaticus* is very rare and it is distributed only in the Eastern Carpathians (Mihál et al. 2003; Rozwałka 2012). It has been found in Slovakia using the leaf litter sieving method in the northeast part of the country within nine grid cells, namely in the geomorphological units (Lehotský et al. 2022): Bukovské vrchy, Ondavská vrchovina, Vihorlatské vrchy, and Východoslovenská pahorkatina (Mašán 1998, 2005; Mihál et al. 2003; Stašiov et al. 2003a; Mihál and Mašán 2006).

**Altitude range and habitat.** The lowest recorded altitude within its known range in Slovakia is 280 m a.s.l.in Bukovské vrchy near Ulič. The highest recorded location is in the National Nature Reserve Stužica, Bukovské vrchy, at 1100 m a.s.l. (Mihál et al. 2003). *Sirocarpaticus* is a forest species that prefers dense and preserved submontane and montane deciduous and mixed forests, particularly beech and oak forests. The species lives primarily in soil and under stones and fallen wood, but it can also be found in detritus, moss, and among decayed rocks (Stašiov 2004).

**Threats and conservation measures.** Among the most common impacts and threats of its population are improper management of forest habitats (e.g., extensive clear-cut logging followed by soil desiccation) – primarily in beech forests, and the planting of tree species that do not occur naturally in the area (e.g., *Pinussylvestris*, *Pinusnigra*, *Larix* sp., *Picea* sp.).

#### ﻿Laniatores Thorell, 1876

**Cladonychiidae Hadži, 1935**

***Holoscotolemonjaqueti* (Corti, 1905)**

*Holoscotolemonjaqueti* (Corti, 1905): 204–226 (Romania, Negreni). Franc (2008), Franc and Mlejnek (1999), Mihál et al. (2009), Stašiov et al. (2003b).

Current distribution in Europe

*Holoscotolemonjaqueti* has a disjunct southeastern European and Carpathian distribution, found in Hungary, Slovakia, Romania, Ukraine, Bosnia and Herzegovina, and Serbia (Raspotnig et al. 2011).

**Current distribution in Slovakia.** This is a rare species recorded in only two grid cells in Slovakia (in the Cerová vrchovina upland). It has a very small population size that cannot be precisely defined, exposing it to an exceptionally high risk of extinction.

**Altitude range and habitat.** It inhabits sub-montane and montane forests, preferring wetter and cooler habitats. Suitable conditions are also found in caves with ample food, high relative humidity, lack of drafts, and temperatures that do not drop below 9 °C year-round. It primarily lives in cave environments, deep talus slopes, or rock crevices. It is found in the upper layers of the soil, detritus, moss, under decaying wood, and stones (Stašiov 2004). In Slovakia, it has been found in Cerová vrchovina upland: National Nature Reserve Pohanský hrad (Labyrintová jaskyňa), Dunivá hora, National Nature Reserve Ragáč (well at Ragáč), and National Nature Reserve Šomoška (alluvium of the Bukovinský stream) (Franc and Mlejnek 1999; Stašiov et al. 2003b; Franc 2008; Mihál et al. 2009).

**Threats and conservation measures.** Factors that may negatively impact *H.jaqueti* populations include tourism, illegal cave entries, and illegal collection by enthusiasts. The cave vestibules where these individuals are active have relatively small areas, which makes the activities of collectors a threat to the small local population. To preserve their habitats, it is essential to refrain from any forestry activities in the immediate vicinity of cave entrances.

#### ﻿Dyspnoi Hansen & Sørensen, 1904

**Nemastomatidae Simon, 1871**

***Paranemastomakochii* (Nowicki, 1870)**

*Nemastomakochi* Nowicki, 1870: 57. Ložek and Gulička (1955), Staręga (1966). Šilhavý 1950: 99–106. Šilhavý (1968a,b), Šilhavý (1974).

*Nemastomakochi*Sørensen (1873), Daday (1918).

*Nemastomaquadripunctatumkochi* Now., Kratochvíl (1933, 1934).

*Nemastomawerneri* Kulczyński (1903), Šilhavý (1974).

*Paranemastomakochii* Nowicki (1870) (mostly Slovakia, High Tatras Mts.), Astaloš (2000), Astaloš and Jarab (2005)Astaloš et al. (1998), Gulička (1985), Hroznár (1981), Kluka and Krumpál (2009), Mihál and Korenko (2010), Maršalek 2004, 2012a,b, 2017, 2018, Maršalek and Stašiov (2015), Mašán and Mihál (1993), Mihál and Astaloš (2011), Mihál and Hrúz (1998), Mihál et al. (2003, 2015), Stašiov (2004), Stašiov et al. (1997, 2003b, 2021), Šilhavý (1970, 1972).

**Current distribution in Europe.** This is a Carpathian endemic species found in Slovakia, the Czech Republic, Poland, Romania, and Ukraine.

**Current distribution in Slovakia.** In the southwestern part of Slovakia, this species has not yet been recorded. Overall, it has been recorded in 58 grid cells. Post-2004, additional records of this species’ occurrence have been contributed by works such as Astaloš and Jarab (2005), Kluka and Krumpál (2009), Mihál and Korenko (2010), Mihál and Astaloš (2011), Maršalek (2004, 2012a, 2012b, 2017, 2018), Maršalek and Stašiov (2015), Mihál et al. (2015), and Stašiov et al. (2021).

**Altitude range and habitat.***Paranemastomakochii* is a hygrophilous montane species that prefers shaded and sufficiently moist habitats without specific preferences for slope exposure. It thrives in forested areas with diverse tree compositions at mid- and higher elevations. Suitable conditions are generally found near springs and streams, in areas with surface seepage, and similar environments. In Slovakia, it prefers mainly beech and fir-beech forests. It has also been found in canyon valleys, peat bogs, spruce forests, riparian vegetation, caves (in the aphotic zone), and other moist habitats. It seeks shelter under stones, decaying wood, moss, and similar substrates (Stašiov 2004).

**Threats and conservation measures.***Paranemastomakochii* is a hygrophilous montane species highly sensitive to environmental changes. Its habitats, including shaded, moist areas near springs and streams, are increasingly degraded due to anthropogenic influences. Key threats include inappropriate forest management, altered hydrological conditions, and water pollution. These activities lead to habitat thinning, soil compaction from heavy logging machinery, and changes in stream quality, often due to pollutants like oil. Effective conservation measures are essential to protect its populations.

***Paranemastomaquadripunctatum* (Perty, 1833)**

*Paranemastomaquadripunctatum* (Perty 1833) (Germany), Bezděčka (2009), Dudich et al. (1940), Šilhavý (1972).

*Paranemastomaquadripunctatumkochi* Now.: Kratochvíl (1934).

*Paranemastomaquadripunctatumwerneri* Kulczyński Kratochvíl (1933, 1934).

Current distribution in Europe

*Paranemastomaquadripunctatum* is a sub-Atlantic to Central European montane species, distributed from eastern France to eastern Poland. Its range extends across northern Germany, Belgium, the Netherlands, the Alpine and Central European countries, and reaches the northern parts of the Balkan and Apennine Peninsulas (Martens 1978).

**Current distribution in Slovakia.** The eastern boundary of its range passes through Slovakia, making it one of the rarest harvestmen species in the country (Stašiov 2003). The oldest records of this species in Slovakia were published by Kratochvíl (1934) from the Tatra Mountains and Turčianske Teplice, Dudich et al. (1940) from the vicinity of Kremnica, and Šilhavý (1972) from the Slovak Karst. These older records are not precisely localised and cannot be assigned to a specific geographical point. Therefore, we omitted these records and did not mark them on the distribution map of *P.quadripunctatum*. The occurrence of this species in Slovakia was later confirmed by Bezděčka (2009) from the Stará Turá locality in the White Carpathians. The most recent record in Slovakia was made by P. Gajdoš and P. Purgat in 2021 in the cadastral area of Ťapešovo, within the Orava Basin (Litavský unpubl.). So, in Slovakia this species is officially confirmed in two grid cells.

**Altitude range and habitat.***Paranemastomaquadripunctatum* prefers shaded and moist habitats in mixed forests of middle and higher elevations. It is also found at the edge of forests and occasionally penetrates shrub areas of open landscapes. It has been recorded in caves as well. It occurs from lowlands to mountainous areas but prefers altitudes ranging from 400 to 1200 metres (Martens 1978).

**Threats and conservation measures.***Paranemastomaquadripunctatum* is a rare, hygrophilous species found in mid- to high-elevation areas, highly sensitive to environmental changes. It serves as an indicator of undisturbed and ecologically valuable environments that provide suitable conditions for various protected and endangered flora and fauna species. Currently, anthropogenic impacts are leading to the degradation of habitats on which this species depends. The main threats of high or medium intensity include improper forest management practices and changes in landscape hydrological conditions. These impacts result in the clearing or removal of forest habitats, alteration of their species and spatial structure, soil compaction due to the use of heavy machinery during logging, and changes in the water regime and quality of streams.

#### ﻿Ischyropsalididae Simon, 1879

***Ischyropsalismanicata* Koch, 1869**

*Ischyropsaliscarli* Lessert (1905), Dudich et al. (1940), Šilhavý (1956).

*Ischyropsalisdacica* Roewer, 1916: 90–158. Ložek and Gulička (1955), Šilhavý (1950, 1956).

*Ischyropsalishelvetica* Roewer (1916), Dudich et al. (1940).

*Ischyropsalishelveticamilleri*Kratochvíl (1933), Kratochvíl (1934).

*Ischyropsalishellwigii* Panzer (1794), Kolosváry (1929), Kratochvíl (1934), Šilhavý (1956).

*Ischyropsalismanicata* Koch (1869) (Romania, Transylvania), Astaloš (2000, 2002), Astaloš and Mihál (2009), Astaloš et al. (1998), Gulička (1985), Hroznár (1981), Košel (1984), Kováč et al. (2007), Kratochvíl (1933, 1934), Maršalek (2012b), Mihál and Hrúz (1998), Mihál and Korenko (2010), Mihál et al. (2003, 2015), Melega et al. (2022), Roewer (1923). Roušar (1999), Staręga (1966), Stašiov (1999, 2004), Stašiov and Bitušík (2001), Stašiov et al. (1997), Stašiov et al. (2003b), Šilhavý (1956, 1968b, 1972, 1974).

*Ischyropsalismilleri*Kratochvíl (1933), Prantl and Mařan (1957).

**Current distribution in Europe.***Ischyropsalismanicata* is a Carpathian endemic species found in Slovakia, the Czech Republic, Poland, Romania, and Ukraine.

**Current distribution in Slovakia.** In Slovakia, this species is moderately common. It is primarily distributed in the northern part of central Slovakia, the Slovak Karst, and the eastern tip of the country. To date, it has been recorded in 50 grid cells. Post-2004 research contributing to the distribution records includes works by Kováč et al. (2007), Astaloš and Mihál (2009), Maršalek (2012b), Mihál and Korenko (2010), Mihál et al. (2015), and Melega et al. (2022).

**Altitude range and habitat.** This harvestman is a hygrophilous mountain species that prefers moist and shaded habitats. It is mainly found in forested mountain areas, often near streams and springs. In Slovakia, it has also been recorded in caves, occupying both the dysphotic and aphotic zones. Outside of caves, it is primarily found in spruce forests, fir-beech-spruce forests, fir-beech forests, beech forests, and dwarf pine areas. It is less commonly found in open habitats such as alpine and moist meadows. This species lives in detritus, moss, under stones, wood fragments, and in the cavities of decaying stumps (Stašiov 2004).

**Threats and conservation measures.***Ischyropsalismanicata* is a hygrophilous mountain species that inhabits shaded habitats near streams and springs, as well as cave environments. Significant negative anthropogenic impacts and threats to its populations include improper forest management and changes in the hydrological conditions of the landscape. The primary threats are habitat loss due to logging, environmental pollution, natural disasters, climate change, and similar factors.

***Gyastitanus* Simon, 1879**

*Gyasannulatus* Olivier (1791), Astaloš (1993, 2000), Dudich (1928), Dudich et al. (1940), Gulička (1985), Hroznár (1981), Kolosváry (1929), Kratochvíl (1933. 1934), Lác (1990), Mašán and Mihál (1993), Staręga (1966), Stašiov (2004), Stašiov et al. (2003b), Šilhavý (1950, 1956, 1968 a,b, 1972, 1974).

*Gyastitanus* Simon (1879), (France), Astaloš (2000), Astaloš et al. (1998), Mihál (1996, 1998), Mihál and Hrúz (1998), Mihál et al. (2003), Stašiov and Mihál (2001).

**Current distribution in Europe.***Gyastitanus* is a European species found in Portugal, Spain, France, Switzerland, Austria, Germany, the Czech Republic, Poland, Hungary, Romania, Italy, Slovenia, Croatia, Bosnia and Herzegovina, Montenegro, Serbia, Slovakia, and Ukraine (Novak et al. 2000; Stašiov 2004).

**Current distribution in Slovakia.** In Slovakia, this species is moderately common in suitable habitats, avoiding extensive lowland areas such as the Danubian lowland and the eastern Slovak lowland. It has been recorded in 48 grid cells. Post-2004 research that expanded the records of this species is presented in works by Astaloš and Jarab (2005), Mihál and Korenko (2010), Maršalek (2004, 2012b, 2017, 2018), Maršalek and Stašiov (2015), and Mihál et al. (2015).

**Altitude range and habitat.***Gyastitanus* is a hygrophilous harvestman species that thrives in shaded, humid mountain environments. It predominantly inhabits mixed moist forests, particularly beech, fir-beech, fir-spruce-beech, and spruce forests in Slovakia, often near streams. This species avoids lowland areas and anthropogenic habitats, favouring moist microhabitats such as rock crevices, moss, fallen wood, and old stumps. Additionally, it has been observed in canyon-like valleys and caves, occupying both dysphotic and aphotic zones (Stašiov 2004).

**Threats and conservation measures.***Gyastitanus* is a moderately common hygrophilous mountain species sensitive to environmental changes. Its habitats, characterised by shaded areas with high humidity and stable temperatures, are currently threatened by intense anthropogenic influences. Key threats to local populations include improper forest management and changes in hydrological conditions, exacerbated by deforestation and climate change. These factors lead to habitat degradation, which significantly affects the survival of the species.

### ﻿The current status of the protection of harvestmen in Slovakia

Although harvestmen are a significant group of animals, their effective protection in Slovakia was not ensured until 2003. None of the species belonging to the order Opiliones were included in the Red Data Book of threatened and rare plant and animal species of the Czech and Slovak Federal Republic 3. Invertebrates published in 1992 (Škapec et al. 1992). Harvestmen were also not included in the Red List of plants and animals of Slovakia published in 2001 (Baláž et al. 2001). Later, some species of harvestmen were included in the list of protected animal species (species of national environmental significance) in Annex 6 of the Decree of the Ministry of the Environment of the Slovak Republic No. 24/2003 Coll., implementing Act No. 543/2002 Coll. on nature and landscape protection, as amended (Ministry of the Environment of the Slovak Republic 2003). This included the following species: *Egaenusconvexus*, *Gyastitanus*, *Ischyropsalismanicata*, *Opiliodinaricus*, *Platybunuspallidus*, and *Sirocarpaticus*. The currently valid regulation is the Ministry of Environment of the Slovak Republic Decree No. 170/2021 Coll. (effective from January 1, 2023), implementing Act No. 543/2002 Coll. on Nature and Landscape Protection, as amended by subsequent regulations. In table 5 of this Decree, the list of protected animal species includes the following species: *Opiliodinaricus*, *Holoscotolemonjaqueti*, *Carinostomaelegans*, *Leiobunumlimbatum*, and *Sirocarpaticus* (Ministry of Environment of the Slovak Republic 2021).

Many European countries have long-established and regularly updated Red Lists of harvestmen, such as Germany (Muster et al. 2016), Austria (Komposch 2009), the Czech Republic (Bezděčka and Bezděčková 2017), and Poland (Głowaciński 2002), among others. Among other reasons, this was also one of the motivations for preparing the first Red List of harvestmen in Slovakia.

Although *Leiobunumlimbatum* has so far been recorded in only six locations in Slovakia (Bezděčka and Bezděčková 2011; Stašiov and Tuf 2016; Stašiov and Diviaková 2022; Litavský unpubl.), we have classified this species under the category LC (Least Concern). The reason is that *L.limbatum* can be considered a species, which primary habitats are rocks and rocky outcrops in forests. Currently, it inhabits various types of habitats.

We included two species of harvestmen in the Data Deficient category. Occurrence of *Opiliodinaricus* has been reliably documented only at two localities in the Vihorlat Mountains (Šilhavý 1966, 1968a). The second harvestman is the genus *Dicranopalpus* sp. In Slovakia, only juvenile individuals from the genus *Dicranopalpus* have been found so far, and they were reliably identified only to the genus *Dicranopalpus*. So far, this genus has only been recorded in Slovakia at two locations, situated in the Vtáčnik Mountains and in the High Tatras (Stašiov 2004).

We have classified *Laciniusdentiger*, *Laciniushorridus*, *Platybunuspallidus*, and *Opilioparietinus* as Near Threatened, as they become increasingly rare over time. It is likely that *Opilioparietinus* is gradually being displaced from its original habitats by *Opiliocanestrinii*, which is more frequently observed in Slovakia. In this matter, Bliss et al. (1996) had previously mentioned this in the Red List of harvestmen of Germany. Since *Opiliocanestrinii* is considered a non-native invasive species in Slovakia, we have classified it under the category NE (Not Evaluated).

Given the ongoing climate change, increasingly intense negative human impact on the environment, as well as the penetration of invasive species into our territory, which suppress populations of our native species (e.g., *Opiliocanestrinii*), it can be expected that the current Red List of proposed harvestman species will need to be updated in the near future.

## ﻿Conclusions

By applying the IUCN Red List criteria, we have identified several species of harvestmen that are at varying levels of endangerment, including one critically endangered species, *Holoscotolemonjaqueti*, and several others that are endangered or vulnerable in Slovakia. The inclusion of these species in a Red List for the first time highlights the urgent need for targeted conservation measures to protect both the species themselves and the habitats they depend on. This checklist and the Red List can serve as foundational tools for conservation planning and policy making in Slovakia, paving the way for more effective protection of this important group of terrestrial invertebrates.

## ﻿Additional information

### Author ORCIDs

Juraj Litavský https://orcid.org/0000-0003-1229-1098

Slavomír Stašiov https://orcid.org/0000-0002-4914-4465

Ivan Mihál https://orcid.org/0000-0003-4582-0750

Martin Šalkovič https://orcid.org/0000-0001-7300-2603

### Data availability

All of the data that support the findings of this study are available in the main text.
